# N2‐Polarized Neutrophils Guide Bone Mesenchymal Stem Cell Recruitment and Initiate Bone Regeneration: A Missing Piece of the Bone Regeneration Puzzle

**DOI:** 10.1002/advs.202100584

**Published:** 2021-08-11

**Authors:** Bolei Cai, Dan Lin, Yan Li, Le Wang, Jirong Xie, Taiqiang Dai, Fuwei Liu, Mingyue Tang, Lei Tian, Yuan Yuan, Liang Kong, Steve G. F. Shen

**Affiliations:** ^1^ Department of Oral & Cranio‐Maxillofacial Surgery Shanghai Ninth People's Hospital College of Stomatology Shanghai Jiao Tong University School of Medicine National Clinical Research Center for Oral Diseases Shanghai Key Laboratory of Stomatology & Shanghai Research Institute of Stomatology Shanghai 200011 China; ^2^ State Key Laboratory of Military Stomatology & National Clinical Research Center for Oral Diseases & Shaanxi Key Laboratory of Oral Diseases Department of Oral and Maxillofacial Surgery School of Stomatology The Fourth Military Medical University Xi'an 710032 China; ^3^ State Key Laboratory of Military Stomatology & National Clinical Research Center for Oral Diseases & Shaanxi Key Laboratory of Oral Diseases Department of Prosthodontics School of Stomatology The Fourth Military Medical University Xi'an 710032 China; ^4^ Department of Prosthodontics School of Stomatology the Jiamusi University Jiamusi 154003 China; ^5^ Key Laboratory for Ultrafine Materials of Ministry of Education School of Materials Science and Engineering and Engineering Research Center for Biomedical Materials of Ministry of Education East China University of Science and Technology Shanghai 200237 P. R. China; ^6^ Shanghai University of Medicine and Health Sciences Shanghai 201318 P. R. China

**Keywords:** bone regeneration, interleukin‐8, neutrophils, stem cell recruitment, stromal cell‐derived factor‐1*α*

## Abstract

The role of neutrophils in bone regeneration remains elusive. In this study, it is shown that intramuscular implantation of interleukin‐8 (IL‐8) (commonly recognized as a chemotactic cytokine for neutrophils) at different levels lead to outcomes resembling those of fracture hematoma at various stages. Ectopic endochondral ossification is induced by certain levels of IL‐8, during which neutrophils are recruited to the implanted site and are N2‐polarized, which then secrete stromal cell‐derived factor‐1*α* (SDF‐1*α*) for bone mesenchymal stem cell (BMSC) chemotaxis via the SDF‐1/CXCR4 (C‐X‐C motif chemokine receptor 4) axis and its downstream phosphatidylinositol 3'‐kinase (PI3K)/Akt pathway and *β*‐catenin‐mediated migration. Neutrophils are pivotal for recruiting and orchestrating innate and adaptive immunocytes, as well as BMSCs at the initial stage of bone healing and regeneration. The results in this study delineate the mechanism of neutrophil‐initiated bone regeneration and interaction between neutrophils and BMSCs, and innate and adaptive immunities. This work lays the foundation for research in the fields of bone regenerative therapy and biomaterial development, and might inspire further research into novel therapeutic options.

## Graphical abstract

Following inflammatory stimuli at the bone defect site, neutrophils arrive first, recruit and orchestrate innate and adaptive immunocytes and BMSCs to initiate endochondral ossification under the appropriate immune environment. In this process, N2‐polarized neutrophils mediate anti‐inflammatory transformation of macrophages and CD4^+^ T cells, secrete SDF‐1*α* for BMSC chemotaxis via the SDF‐1/CXCR4 axis and its downstream PI3K/AKT pathway and *β*‐catenin‐mediated migration.

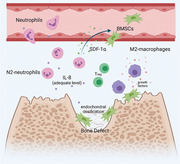

## 1 Introduction

Compared to stem cell transplantation, regenerative therapies based on endogenous stem cell recruitment provide an optimistic outlook for clinical applications.^[^ ^1^ ^]^ Bone mesenchymal stem cells (BMSCs) play important roles in bone regeneration by differentiating into bone‐forming osteoblasts, which further transform into osteocytes, produce bone matrix, and result in increased bone mass.^[^ ^2^ ^]^ With an improved understanding of the cellular microenvironment, cell signaling, and cell‐cell contact, researchers have developed strategies to enhance BMSC recruitment, preserve stem cell function, and guide tissue regeneration.^[^ ^3^ ^]^ However, the biological process of how BMSCs are recruited to specific sites remains elusive, and uncovering this information may greatly improve current regenerative therapies.

After injuries and therapeutic implantation, immune cells trigger inflammation that activates the regenerative cascade.^[^ ^4^, ^5^ ^]^ Among all immunocytes, macrophages have been most widely studied, are considered key players as a key participator in tissue regeneration, and function as regulators of BMSC differentiation,^[^ ^6^ ^]^ while neutrophils are scarcely recognized, except for their proinflammatory and antimicrobial activity,^[^ ^7^ ^]^ and are presumed to be detrimental to bone formation.^[^ ^8^ ^]^ Recent studies revealed the anti‐inflammatory and healing capacities of neutrophils by recruiting monocytes and other immune cells.^[^ ^5^, ^9^ ^]^ A canonical binary classification of proinflammatory neutrophils (N1) and anti‐inflammatory neutrophils (N2) was originally proposed in oncology and is also used in the regenerative immunology field.^[^ ^10^ ^]^ However, the status and function of neutrophils in the bone regenerative process remain elusive.

**Figure 1:**
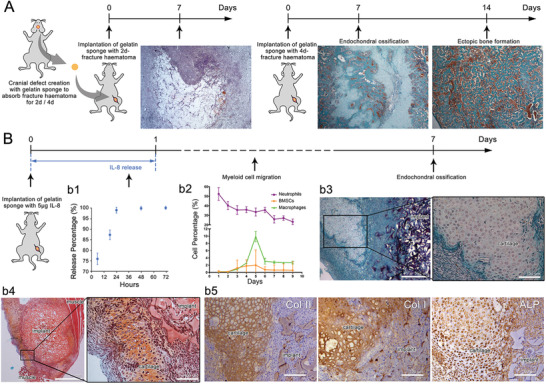
Intramuscular ectopic endochondral ossification induced by fracture hematoma and IL‐8 were similar at the early stage, recruiting neutrophils first and BMSCs and macrophages at a later stage. A) Histological observation of intramuscular implantation of 2‐day and 4‐day cranial defect hematoma using Masson's trichrome staining. 4‐day hematoma‐induced ectopic endochondral ossification. B) b1) In vitro release profile of IL‐8 from gelatin sponge. IL‐8 loaded onto gelatin sponge was rapidly released within 24 h. b2) Time‐course cytometric quantification of myeloid cell subpopulations in the implant using flow cytometry. Neutrophils promptly appeared and peaked at day 1, while BMSCs and macrophages peaked at day 5. Histological observation at day 7 using b3) Masson's trichrome staining and b4) Safranin‐O staining. Ectopic cartilage was formed around the implant. b5) Type II collagen (Col II), type I collagen (Col I), and alkaline phosphatase (ALP) immunohistochemistry staining. The exclusive cartilaginous Col II, together with the osteogenic‐related Col I and ALP indicated ectopic endochondral ossification. (*n* = 3 for each group)

Bone fractures cause cellular damage at the defect site and a local release of cytokines, culminating in a “cytokine storm,” which presents as macroscopic “fracture hematoma”. As early as in 1990, researchers had proven that fracture hematoma was crucial to regeneration, and that the hematoma at specific time point could induce intramuscular ectopic endochondral ossification,^[^ ^11^ ^]^ which was reproduced in this study by implantation of cranial defect hematoma into the thigh muscle pouch of mice (**Figure** **1**A). However, hematoma complexity hindered the exploration of the mechanism underlying regeneration initiation. Our previous study^[^ ^12^ ^]^ unexpectedly revealed induction of intramuscular ectopic endochondral ossification by interleukin‐8 (IL‐8), a known chemotactic cytokine for neutrophils,^[^ ^13^ ^]^ which is reported to be highly expressed at trauma sites.^[^ ^14^, ^15^ ^]^ This phenomenon highlights the critical role of IL‐8 and neutrophils in bone regeneration, and provides a single‐variable model to explore the mechanism of regeneration initiation. In this study, we hypothesize that IL‐8 initiates swarming of neutrophils and release of neutrophil‐derived cytokines, which consequently activate regenerative cascades. Neutrophils, among all immune and non‐immune cells, may play important roles in the upstream cellular processes underlying bone regeneration. Herein, the function of neutrophils in the bone regenerative process is unraveled and discussed.

## 2 Results

### 2.1 Interleukin‐8 Induced Ectopic Endochondral Ossification Resembling Early‐stage Bone Regeneration

As shown in Figure 1A, a cranial defect was created and filled with a commercialized medical absorbable gelatin sponge to absorb the hematoma for 2 or 4 days (identical time points as previous study),^[^ ^11^ ^]^ and was then implanted into the thigh muscle pouch. The 2d‐hematoma/gelatin was largely degraded with many surrounding immunocytes after 7 days, while the 4d‐haematoma/gelatin induced an ectopic bone formation via endochondral ossification, with extensive cartilage formed after 7 days and trabecular bone formation after 14 days, consistent with the findings of the previous research.^[^ ^11^ ^]^

IL‐8 (5 µg) was loaded onto a gelatin sponge via solution dropping and lyophilization. As the in vitro release profile indicated, 99% of the IL‐8 was released within 24 h (Figure 1B,b1). After implantation in thigh muscle pouches of mice, time‐course cellular components on the IL‐8/gelatin implant were detected using flow cytometry to profile three myeloid cell subpopulations: neutrophils (CD45^+^, Ly6G^+^), macrophages (CD45^+^, Ly6G^−^, F4/80^+^), and BMSCs (CD45^−^, CD44^+^, CD29^+^) (Figure 1B,b2). Neutrophils were first recruited to the implantation site and in the largest number, which peaked at day 1, BMSC recruitment occurred from day 2 and peaked at day 4–5, macrophage recruitment started from day 2 and peaked at day 5.

Cartilage formation was observed around the implant seven days post‐implantation, using Masson's trichrome staining (Figure 1B,b3), which was further confirmed by using Safranin‐O staining (Figure 1B,b4), a common method to identify chondrocytes and cartilage matrix. The implant and its surrounding cells were wrapped by a fibrous capsule, forming an isolating microenvironment from the muscular tissues. Immunohistochemistry staining was applied to identify the status of the ectopic cartilage (Figure 1B,b5): The positive expressions of cartilaginous type II collagen (Col II), osteogenic‐related type I collagen (Col I), and alkaline phosphatase (ALP) indicated the tendency of endochondral ossification. However, the IL‐8/gelatin implant was insufficient to induce further ossification and the cartilage/implant was completely adsorbed and degraded after 14 days. Gelatin sponge with a low IL‐8 dose (1 µg) was insufficient to recruit myeloid cells and induce endochondral ossification, while a high IL‐8 dose (10 µg) led to the rapid degradation of the gelatin indicating predominant catabolism (Figure S1, Supporting Information). The results indicated that only an appropriate range of IL‐8 concentrations could induce intramuscular ectopic endochondral ossification.

IL‐8 is commonly recognized as the most potent chemokine for neutrophils^[^ ^16^ ^]^ and is reportedly involved in chemotaxis of BMSCs.^[^ ^17^ ^]^ Herein, neutrophils were recruited immediately after IL‐8 release, while BMSCs arrived much later, implying IL‐8 is a direct chemoattractant for neutrophils, while indirect for BMSCs. From the aspect of cellular component, the endochondral ossification originated from the recruited BMSCs, which are well‐known for their osteogenic differentiation.^[^ ^18^ ^]^ However, the relationship between neutrophil chemotaxis and BMSC recruitment is poorly understood. It is speculated that an appropriate concentration of IL‐8 recruited neutrophils and formed a microenvironment similar to a bone lesion, which further recruited other myeloid cells and promoted the chondrogenic/osteogenic differentiation of BMSCs. Though the microenvironment was temporary, it could be utilized as a simplified model to investigate the interaction between neutrophils and BMSCs during regeneration initiation.

### 2.2 Neutrophils were Essential for Bone Regeneration at the Initiation Stage

The above results indicated that neutrophils may function as initiators in early bone regeneration based on myeloid cell recruitment and endochondral ossification. To verify this possibility, the early‐stage influence of neutrophils and macrophages on critical cranial defect regeneration was compared through depletion by neutralizing antibodies.

**Figure 2:**
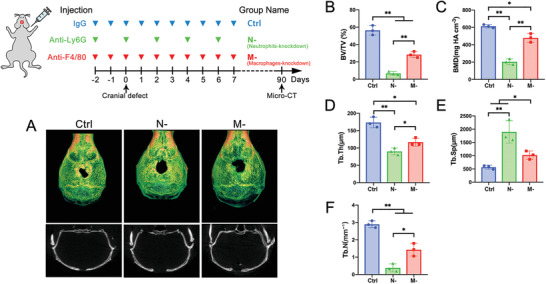
Neutrophils were pivotal for bone‐healing at the initiating stage. *α*‐Ly6G or *α*‐F4/80 antibody was continuously injected from 2 days pre‐ to 7 days post‐cranial defect creation for depletion of neutrophils or macrophages (referred to as N‐ and M‐), respectively, with IgG antibody as a control. A) Micro‐CT reconstruction of the defect site after 90 days. Quantification histogram of B) bone volume/total volume (BV/TV), C) bone mineral density (BMD), D) trabecular thickness (Tb.Th.), E) trabecular space (Tb.Sp.), and F) trabecular number (Tb.N.). (** *p* < 0.01, * *p* < 0.05; *n* = 3 for each group)

As shown in **Figure** **2**, from 2 days before to 7 days after calvarial defect creation, circulatory neutrophils and monocytes (macrophage precursor) were depleted by continuously injecting anti‐Ly6G and anti‐F4/80 neutralizing antibodies, respectively (referred to as N‐ and M‐, with IgG antibody as control). The treatment efficiently and specifically depleted neutrophils or monocytes in peripheral blood without affecting the other cell type (Figure S2, Supporting Information).

Ninety days post‐operation, the defect‐healing status was analyzed using micro‐CT, with bone volume/total volume (BV/TV) as the main indicator of bone healing. Critical defects could not be completely healed by autologous healing capacity. In the control group, new bone formation was observed around the edge of the defect with a BV/TV of 56.33% ± 5.50%; the BV/TV was significantly reduced in the M‐ group (28.3% ± 3.51%) and completely inhibited in the N‐ group (6.6% ± 2.08%). Bone mineralized density (BMD), trabecular thickness (Tb.Th.), and trabecular number (Tb.N.) exhibited similar trends among the three groups, with a consistent opposing trend in trabecular space (Tb.Sp.) (Figure 2B–F).

The fact that early‐stage depletion of circulatory immunocytes significantly impaired the long‐term healing outcome, indicated a critical time window for regeneration initiation, which, if missed, could lead to failure of regeneration. In this specific initiation stage, a lack of macrophages compromised regeneration, while a lack of neutrophils completely incapacitated autologous healing capacity. These results further suggest that neutrophils may initiate or orchestrate the regeneration process.

### 2.3 Interleukin‐8‐Chemotactic Neutrophils Initiated Regenerative Cascade by Recruiting Bone Mesenchymal Stem Cells

The cranial defect, which inherently contained complex cellular components, was too complicated to investigate the regeneration initiation process at the cellular level. Therefore, the IL‐8‐induced intramuscular ectopic endochondral ossification model was applied to investigate the specific role of neutrophils in bone regeneration initiation.

**Figure 3:**
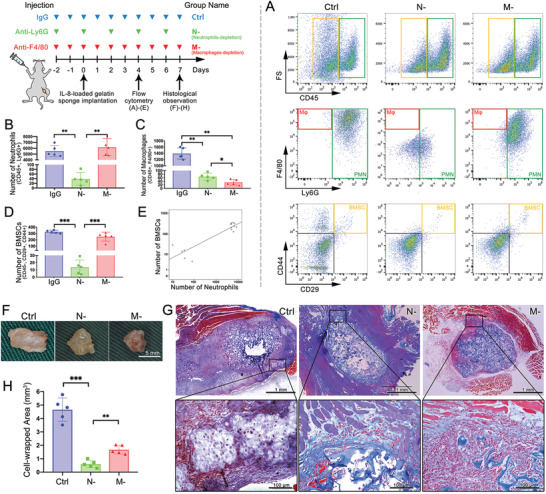
Depletion of circulatory neutrophils cut off the IL‐8‐induced myeloid cell recruitment and ectopic endochondral ossification. A) Flow cytometry of the ectopic implant and its quantitative histogram of B) neutrophils, C) macrophages, and D) BMSCs. Depletion of neutrophils inhibited recruitment of all myeloid cells. E) Numbers of neutrophils and BMSCs exhibited a positive correlation. F) Implants retrieved at day 7, G) histological observation using Masson's trichrome staining, and H) quantification of cell‐enwrapped areas. Control group induced ectopic cartilage formation, while N‐ and M‐ significantly decreased the cell‐wrapped area and prevented further differentiation. (*** *p* < 0.001, ** *p* < 0.01, * *p* < 0.05; *n* = 5 for each group)

Neutralizing antibodies were injected from 2 days pre‐ to 7 days post‐implantation for circulatory neutrophil/monocyte depletion, myeloid cell subpopulations were detected using flow cytometry on day 4, and ectopic endochondral ossification was observed on day 7 (**Figure** **3**). In peripheral blood, the injection depleted the targeted cells without affecting other cell subpopulation (Figure S2, Supporting Information), while in the implant, depletion of neutrophils (N‐ group) significantly reduced the recruitment of both BMSCs and macrophages (Figure 3A–D). In contrast, depletion of monocytes (M‐ group) significantly reduced only the macrophage subpopulation in the implant, without affecting BMSCs and neutrophils compared to the control group. Using Pearson Correlation Coefficient analysis, correlation between the numbers of recruited BMSCs and neutrophils proved to be highly relevant (*r* = 0.82, *p* < 0.01, *n* = 15) (Figure 3E). These results implied that neutrophils acted as upstream participants that recruited other myeloid cells at the initial stage of bone regeneration.

At day 7, the implants were retrieved (Figure 3F) and observed using Masson's trichrome staining (Figure 3G). The cellular area wrapped in the fibrous capsule was quantified (Figure 3H). Compared with the control group, depletion of either monocytes or neutrophils significantly reduced the cell‐wrapped areas and impaired ectopic endochondral ossification. The cell‐wrapped area not only correlated with the number of cells, but also related to the amount of the secreted extracellular matrix (ECM). As shown in Figure 3G, the implant in the control group was surrounded by a large number of cells and ECM of endochondral ossification, which contained Col II and Col I (Figure S3, Supporting Information). The control group exhibited a cell‐wrapped area of 4.7 ± 0.9 mm^2^. The M‐ group displayed a reduced cell recruitment and slight ECM secretion, with a cellular area of 1.7 ± 0.3 mm^2^, while the N‐ group barely exhibited cell recruitment around the implant (0.6 ± 0.3 mm^2^). Immunohistochemistry staining of Col II and Col I (Figure S3, Supporting Information) indicated that either depletion of neutrophils or monocytes significantly undermined chondrogenic and osteogenic expression.

**Figure 4:**
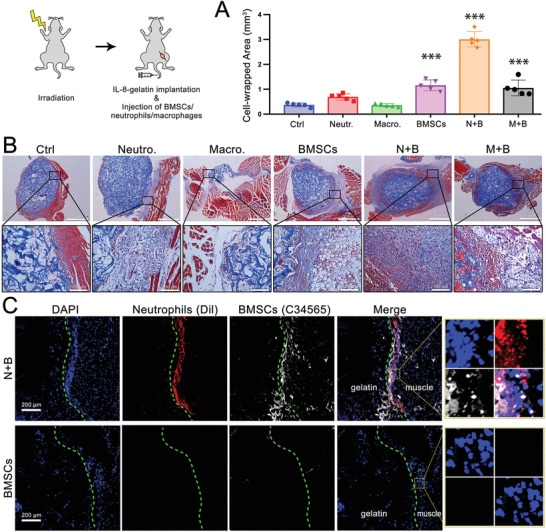
Interaction of neutrophils and BMSCs were key to anabolism, while macrophages were essential for catabolism. Irradiated mice received IL‐8‐gelatin implantation and injection of exogenous myeloid cells. A) Quantification of cell‐enwrapped areas and B) histological observation using Masson's trichrome staining. C) Fluorescent‐labeled neutrophils and BMSCs around the implant. When neutrophils and BMSCs were simultaneously transfused (N+B), greater cell recruitment and ECM were observed around the implant; when only macrophages were transfused the implant was rapidly degraded. (Compared with ctrl: *** *p* < 0.001, ** *p* < 0.01, * *p* < 0.05; *n* = 5 for each group).

To further elucidate the relationship between neutrophils and BMSCs, exogenous myeloid cells were adoptively transferred to irradiated mice (myeloid cell depletion > 90%, Figure S4, Supporting Information). The transferred myeloid cells were extracted and purified from bone marrow of un‐irradiated donor mice (Figure S5, Supporting Information). Three days after nonlethal irradiation, IL‐8/gelatin was implanted intramuscularly, and different components of exogenous myeloid cells were injected intravenously to observe cell recruitment around the implant (**Figure** **4**).

As shown in Figure 4A,B, without injection of exogenous myeloid cells (Ctrl), sparsely scattered cells were recruited around the implant, and were considered as the residual myeloid cells in irradiated mice (Ctrl, cell‐wrapped area of 0.4 ± 0.1 mm^2^). Neutrophil‐transfer exhibited a cluster of cells wrapped around the implant with slight ECM secretion (0.7 ± 0.1 mm^2^). Monocyte‐transfer resulted in reduced cell recruitment (0.4 ± 0.1 mm^2^), but significantly degraded gelatin, which was attributed to macrophage phagocytosis. BMSC‐transfer presented increased cell recruitment and a considerable number of adipose vacuoles (1.1 ± 0.2 mm^2^), indicating adipogenic differentiation instead of chondrogenic/osteogenic differentiation. Transfer of monocytes and BMSCs (M+B) exhibited cell recruitment to a certain extent but with little ECM secretion (1.4 ± 0.3 mm^2^). Among all groups, transfer of neutrophils and BMSCs (N+B) exhibited the largest cell‐wrapped area (3.0 ± 0.3 mm^2^) with extensive cell recruitment and secretion of collagen‐containing ECM (stained blue with Masson's trichrome stain), which may indicate a tendency for chondrogenic/osteogenic differentiation. Nonetheless, transfer of neutrophils and BMSCs could not restore the IL‐8‐induced ectopic endochondral ossification at day 7, since injection of exogenous myeloid cells could not compare with the inherent bone marrow‐derived cells, either in cell number or in cellular component. By comparing the BMSC‐transfer group with the N+B‐transfer group, it was inferred that neutrophils not only participated in BMSC recruitment, but also influenced their differentiation.

To further identify the cellular component of the IL‐8/gelatin‐recruited cells, before intravenous injection, the purified neutrophils and BMSCs were labeled with fluorescent cell‐tracker probes Dil (red) and C34565 (white), respectively. As shown in Figure 4C, two days after implantation and transplantation, N+B transfer revealed that a large number of cells tightly surrounded the implant, consisting of the labeled neutrophils and BMSCs, while BMSC‐transfer presented few recruited BMSCs. The result further confirmed that IL‐8‐recruited neutrophils were a prerequisite for the subsequent recruitment of BMSCs from blood circulation, which explains the relevance between the recruitment of BMSCs and neutrophils (Figure 3E).

In summary (Figures 1, 2, 3, 4), after bone injury, with inflammatory cytokines secreted at the defect site (typically, IL‐8), neutrophils arrived first and recruited BMSCs and macrophages, then macrophages regulated the fate of BMSCs toward chondrogenic and osteogenic differentiation, which macroscopically presented as endochondral ossification. In this process, neutrophils, macrophages, and BMSCs were considered as initiators, mediators, and direct participants of bone regeneration, respectively.

### 2.4 N2‐Polarized Neutrophils under Adequate Interleukin‐8 Concentration and Secreted Stromal Cell‐Derived Factor‐1*α* to Recruit Bone Mesenchymal Stem Cells

**Figure 5:**
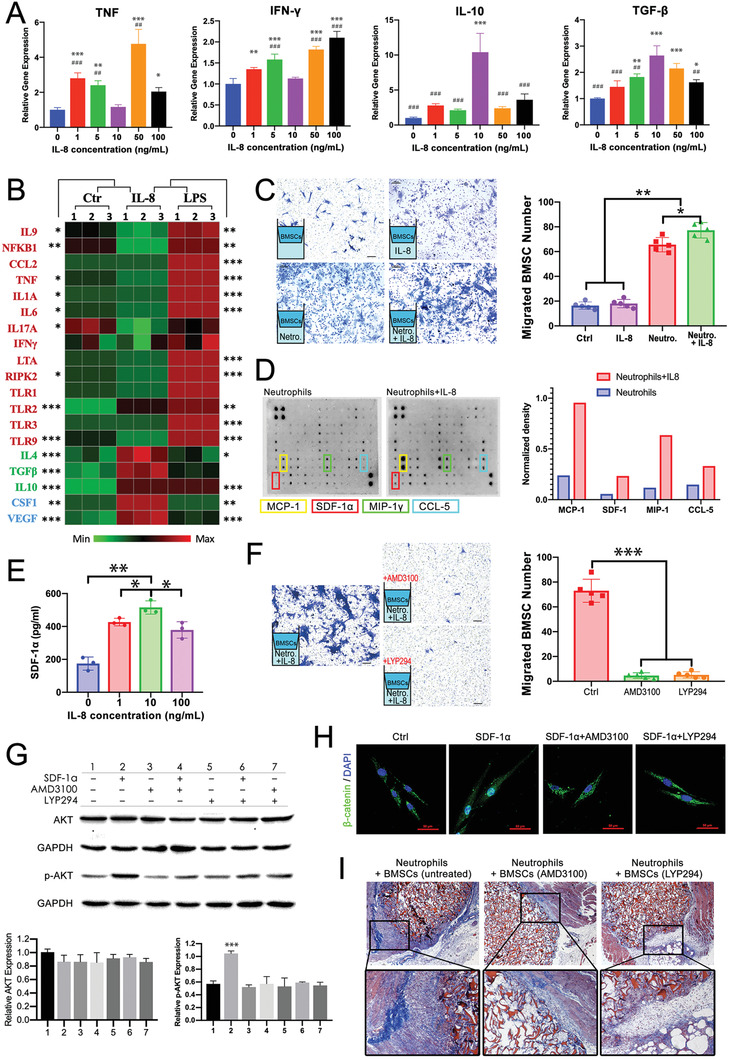
IL‐8 (10 ng mL^−1^) polarized neutrophils toward the N2 phenotype, which secreted SDF‐1*α* to mediate BMSC recruitment and differentiation via the SDF‐1/CXCR4 axis and its downstream PI3K/AKT pathway and *β*‐catenin‐mediated migration. A) Expressions of proinflammatory and anti‐inflammatory genes in neutrophils under different concentrations of IL‐8. B) Heat map of mouse inflammatory response and autoimmunity profiler PCR array: Untreated (Ctr), IL‐8‐, and LPS‐treated neutrophils (10 ng mL^−1^, 24 h exposure). Data are row‐relative and normalized to GAPDH. C) Transwell assay highlighting BMSC recruitment by IL‐8, neutrophils, and IL‐8‐treated neutrophils (migrated cells counted in five random 200 × microscopic fields). D) Mouse cytokine antibody array analyses of conditioned media from neutrophils or IL‐8‐treated neutrophils (10 ng mL^−1^, 24 h exposure) and the top four upregulated cytokines quantified using densitometry. E) ELISA‐determined SDF‐1*α* secreted by neutrophils treated with IL‐8 at different concentrations. F) Recruitment of BMSC pretreated with CXCR4 antagonist AMD3100 and PI3K/AKT inhibitor LY294002. G) SDF‐1‐induced phosphorylation of Akt was inhibited by AMD3100 or LY294002. H) Immunofluorescent staining of *β*‐catenin translocated to nuclei by SDF‐1*α* and inhibited by AMD3100 or LY294002. I) Irradiated mice received IL‐8‐gelatin implantation and injection of exogenous neutrophils and BMSCs pretreated with AMD3100 or LY294002. (Compared with ctrl: *** *p* < 0.001, ** *p* < 0.01, * *p* < 0.05. Compared with 10 ng mL^−1^: ### *p* < 0.001, ## *p* < 0.01, # *p* < 0.05; *n* = 3 for each group in PCR and ELISA; *n* = 5 for each group in transwell assay).

Since IL‐8 dosage influenced the intramuscular implantation outcome, expressions of typical inflammatory genes in neutrophils under various IL‐8 concentrations were evaluated. As shown in **Figure** **5**A, expression of the anti‐inflammatory interleukin‐10 (IL‐10) and transforming growth factor‐*β* (TGF‐*β*) peaked in response to IL‐8 at a moderate concentration of 10 ng mL^−1^, while proinflammatory tumor necrosis factor (TNF) and interferon‐*γ* (IFN‐*γ*) were predominantly upregulated at higher concentrations (50 and 100 ng mL^−1^). Results observed in vitro resembled those of the in vivo model, in which only a medium dosage of IL‐8 (5 µg) resulted in ectopic osteochondral ossification.

The IL‐8 concentration (10 ng mL^−1^) for N2‐polarization of neutrophils was further applied in following in vitro experiments. In comparison, lipopolysaccharide (LPS), which is a component of various gram‐negative bacteria and is a common inflammatory stimulus for neutrophils, was applied to induce N1‐polarization at an identical concentration. Correspondingly, a gelatin sponge loaded with 5 µg LPS was implanted into thigh muscle pouches of mice, and after 7 days the gelatin was totally degraded and pathological fat liquefaction was observed at the implant site (Figure S1, Supporting Information).

Mouse inflammatory response and autoimmunity profiler PCR array was conducted to further detect the status of neutrophils polarized by the specific IL‐8 concentration (Figure 5B). IL‐8‐treated neutrophils exhibited an opposite phenotype to LPS‐treated neutrophils, with lower expression of canonical proinflammatory genes (IL‐9, NFKB1, CCL2, CCL5, TNF, IL‐1A, IL‐6, IL‐17A, IFN‐*γ*, LTA, RIPK2, TLR1, TLR2, TLR3, TLR9) and higher expression of anti‐inflammatory (IL4, TGF‐*β*, IL10) and pro‐remodeling genes (CSF1, VEGF*α*), confirming the N2‐polarization at 10 ng mL^−1^ IL‐8 concentration. Notably, downregulation of CCL2, CCL5, IL‐6, IL‐17A, and IFN‐*γ* and upregulation of TGF‐*β* may be related to the transformation of CD4^+^ T cells from proinflammatory Th1 and Th17 to anti‐inflammatory T_reg_ cells.^[^ ^19^ ^]^

The results showed that IL‐8 at a moderate level led to anti‐inflammatory N2‐polarization of neutrophils and ectopic endochondral ossification, indicating that N2‐polarized neutrophils play a crucial role in endochondral ossification‐based bone regeneration. However, high concentrations of IL‐8 led to proinflammatory N1‐polarized neutrophils and degradation of the implanted material. As a typical inflammatory factor, IL‐8 level at the defect site is time‐dependent and associated with the phases of wound healing. At different stages of bone regeneration, neutrophils, in analogy to the M1 and M2 paradigm of macrophages, are polarized into N1 (proinflammatory) or N2 (anti‐inflammatory) phenotypes by phasic microenvironment of inflammatory factors,^[^ ^20^ ^]^ and then in turn mediate the immunologic defense or wound healing accordingly. It was inferred that ectopic implantation of IL‐8 at various concentrations simulated a bone defect microenvironment at different stages, thus resulting in different outcomes (Figure S1A, Supporting Information).

In vitro chemotaxis transwell assay was applied as a single variable model to further investigate the interaction among IL‐8, neutrophils, and BMSCs. As shown in Figure 5C, IL‐8 itself barely exerted chemotaxis on BMSCs compared to the control group, while neutrophils exhibited remarkable capacity for BMSC recruitment, which increased significantly after neutrophils were treated with IL‐8 (10 ng mL^−1^). Additionally, wound‐healing assays suggested that IL‐8‐treated neutrophils significantly enhanced the migration of BMSCs (Figure S6, Supporting Information). The transwell assays, as indirect co‐culture systems without cell‐cell contact, indicated that neutrophils recruited and mediated BMSCs by releasing biological signals, which was enhanced by IL‐8 treatment.

To gain further insight, a mouse cytokine antibody array was applied to evaluate the secreted chemokines by IL‐8‐treated and ‐untreated neutrophils in serum‐free supernatant. The top four upregulated cytokines, post‐IL‐8 treatment, were marked in Figure 5D: Monocyte chemoattractant protein‐1 (MCP‐1, also known as CCL2), macrophage inflammatory protein‐1*γ* (MIP‐1*γ*), CC chemokine ligand 5 (CCL5), and stromal cell‐derived factor‐1*α* (SDF‐1*α*). Among which, MCP‐1, MIP‐1*γ*, and CCL5 were reported to be regulators and chemoattractants for immunocytes, including monocytes, macrophages, and T cells,^[^ ^21^, ^22^ ^]^ while SDF‐1*α* is known as a highly potent chemoattractant for MSC migration from stem cell niche to injury site,^[^ ^23^ ^]^ and also participated in endogenous endochondral ossification.^[^ ^24^ ^]^ As shown in Figure 5E, SDF‐1*α* secretion levels of IL‐8‐treated neutrophils peaked at 10 ng mL^−1^, indicating that the IL‐8 concentration for N2‐polarization of neutrophils was also optimal for BMSC recruitment. Therefore, it is highly possible that SDF‐1*α* was a key element in the process of IL‐8‐polarized neutrophils inducing ectopic endochondral ossification.

High expression of SDF‐1*α* has been reported in various injured tissue types, including bone fracture, interacting with its specific receptor CXCR4 to direct the migration of stem cells to the injury site via chemoattractant gradient.^[^ ^23^, ^25^ ^]^ Studies have suggested that the SDF‐1/CXCR4 axis may mediate BMSC migration via activation of the phosphatidylinositol 3'‐kinase (PI3K)/Akt signaling pathway.^[^ ^26^ ^]^ BMSCs were pretreated with CXCR4 antagonist (AMD3100) or PI3K/Akt inhibitor (LY294002) before the transwell assay, and both pretreatments significantly repressed BMSC migration toward IL‐8‐treated neutrophils (Figure 5F), confirming that neutrophils recruited BMSCs via the SDF‐1/CXCR4 axis and PI3K/Akt pathway.

Signal transition induced by SDF‐1*α* in BMSCs was investigated by detecting phosphorylation levels of Akt in untreated and AMD3100‐ and LY294002‐pretreated BMSCs using western blot (Figure 5G). SDF‐1*α* evidently activated the phosphorylation of Akt, which was inhibited by pretreatment with AMD3100 or LY294002, indicating that PI3K/Akt served as the downstream pathway of the SDF‐1/CXCR4 axis. Additionally, *β*‐catenin, a multifunctional protein that mediates cell skeleton rearrangement and cell migration, was also demonstrated as a downstream signaling molecule of SDF‐1*α* stimulation (Figure 5H). *β*‐catenin, located at the cell surface, links to actin cytoskeleton, and when activated, dissociates from cell membrane and translocates into the nucleus to regulate the expression levels of cytoskeleton proteins.^[^ ^27^ ^]^ As shown in Figure 5H, untreated BMSCs exhibited distributed membranous expression of *β*‐catenin (Ctrl), which was significantly upregulated and translocated to nuclei by SDF‐1*α*; while pretreatment of AMD3100 or LY294002 inhibited the nuclear translocation of *β*‐catenin. The results demonstrated that SDF‐1*α*/CXCR4 interaction activated the PI3K/AKT signaling pathway and consequently led to nuclear translocation of *β*‐catenin for cell migration.

Further, the critical role of SDF‐1/CXCR4 and its downstream PI3K/Akt axis was corroborated in the IL‐8‐induced endochondral ossification process in vivo. Neutrophils, together with untreated and AMD3100‐ or LY294002‐pretreated BMSCs, were transferred into irradiated mice as exogenous myeloid cells from 2 days pre‐ to 7 days post‐implantation of IL‐8/gelatin in thigh muscle pouches. As shown in Figure 5I, BMSC pretreatment by AMD3100 and LY294002 both significantly reduced the cell‐wrapped area around the implantation compared to untreated BMSCs. These results verified that the in vivo recruitment of BMSCs by neutrophils also occurred via SDF‐1/CXCR4 interaction and downstream PI3K/Akt signaling.

## 3 Discussion

In this study, the role of neutrophils and their interaction with BMSCs in the initiation process of bone regeneration was first elucidated through in vivo orthotopic bone regeneration experiments and ectopic simulation of endochondral ossification to in vitro cell behavior and molecular evaluations. Orthotopic bone defects display complex microenvironments with many signaling molecules (such as, cytokines and enzymes) and cell types (including both tissue resident myeloid cells and those recruited via circulation), and are therefore unsuitable as in vivo models for studying the intercellular interaction during bone regeneration. On the contrast, intramuscular ectopic models have been commonly applied as a simplified and simulative in vivo model for osteogenic‐related investigation.^[^ ^28^ ^]^

**Figure 6:**
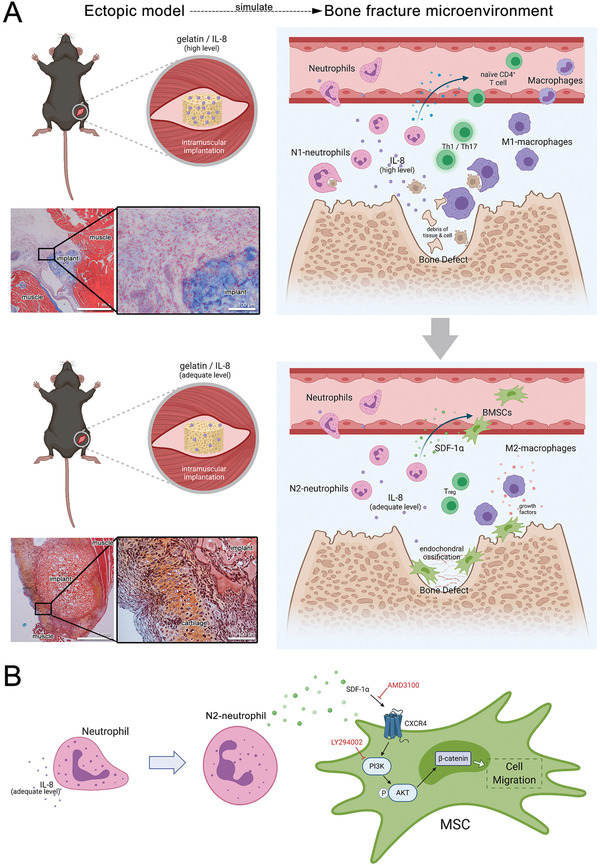
A) The role of neutrophils in the initiation process of bone regeneration elucidated via in vivo ectopic simulation of endochondral ossification. The ectopic implantation of IL‐8 at a high and medium level have simulated the microenvironment of bone fracture at the early and later stage, respectively. High‐level IL‐8 at the early stage led to a proinflammatory microenvironment and phagocytosis to eliminate the damaged tissue debris for inflammation resolution. Thereafter, an adequate level of IL‐8 at the later stage polarizes neutrophils to an N2‐subtype for endochondral ossification. B) Signaling pathway of BMSC recruitment by neutrophils. N2‐polarized neutrophils release SDF‐1*α* to recruit BMSCs via the SDF‐1/CXCR4 axis, its downstream PI3K/Akt pathway, and *β*‐catenin‐mediated migration.

In the ectopic model of endochondral ossification (Figure 1B and Figure S1, Supporting Information), high‐level and medium‐level IL‐8, led to entirely different outcomes. As reported previously^[^ ^11^ ^]^ and determined in this study (Figure 1A), fracture hematoma at different time points also led to entirely different outcomes when implanted at ectopic sites. The pattern could be connected with the time‐dependent cytokine level of bone fractures, as another study reported that IL‐8 level after fracture was first elevated then decreased.^[^ ^14^ ^]^ Therefore, it is presumed that the ectopic implantation of high‐level and medium‐level IL‐8 simulate the microenvironment of bone fracture at early and late stages, respectively (**Figure** **6**A).

Bone regeneration is the result of interaction between various cell types. However, mechanisms underlying initiation of bone regeneration post‐injury remain inconclusive. Though this study focused on the interaction between neutrophils and BMSCs, it was observed that macrophages also play an important role in bone regeneration (Figure 2), and that there is a potential interaction between neutrophils and T cells (Figure 5B). Similar to previous studies, the findings of this study provide information on a part of the bigger puzzle, and the research on this taken together can be used to delineate a possible mechanism of bone regeneration initiation (Figure 6A).

### 3.1 Inflammatory Phase

Shortly after injury, cell debris and neighboring cells at the defect site release massive inflammatory signals. Neutrophils, consisting 10–25% of the circulating leukocytes in mice and 50–70% in humans, are usually the first and most abundant immunocytes to arrive at the defect site.^[^ ^29^ ^]^ In this microenvironment with a high level of inflammatory signals, neutrophils recruited from the circulatory system are N1‐polarized (proinflammatory subtype) and secrete cytokines that recruit other types of immunocytes. At the same time, ECM protein fibronectin, which provides structural support for cell adhesion^[^ ^30^ ^]^ and tissue remodeling during wound healing,^[^ ^31^ ^]^ acts as a key modulator of degranulation and amplifies matrix metalloproteinase (MMP) release by neutrophils.^[^ ^32^ ^]^ ECM components, including fibronectin, as known MMPs substrates, are enzymatically hydrolyzed by N1‐neutrophil secretions. Meanwhile, N1‐neutrophils also synthesize new fibronectin to remodel peripheral tissue for entrance of other somatic cells.^[^ ^32^, ^33^ ^]^

N1‐neutrophils produce cytokines to recruit monocyte‐derived macrophages, including Annexins,^[^ ^34^, ^35^ ^]^ MMPs,^[^ ^35^ ^]^ monocyte chemoattractant proteins (MCPs),^[^ ^36^ ^]^ and macrophage inflammatory proteins (MIPs).^[^ ^37^ ^]^ In this proinflammatory microenvironment, the macrophages are M1‐polarized to remove tissue debris and apoptotic neutrophils to resolve inflammation and prepare the microenvironment for bone regeneration.^[^ ^5^, ^29^ ^]^

Meanwhile, N1‐neutrophils recruit Th17 cells via production of CCL2 and CCL20 chemokines and Th1 cells via production of CCL2, CXCL9, and CXCL10.^[^ ^38^ ^]^ The newly recruited Th17 cells are induced to produce IL‐17 after cell–cell interactions with the inflammation‐activated M1‐monocytes.^[^ ^39^ ^]^ Notably, N1‐neutrophils and M1‐macrophages also produce IL‐17 (Figure 5B).^[^ ^40^ ^]^ In turn, IL‐17, as a proinflammatory cytokine, stimulates epithelial cells to secrete CXC chemokines including CXCL8 (IL‐8) and G‐CSF, which amplify neutrophil recruitment and activation.^[^ ^41^, ^42^ ^]^ IL‐17 can also stimulate the expression of CCL2 (MCP‐1) and CCL20 (MIP‐3*α*) to amplify monocyte recruitment.^[^ ^41^ ^]^ This process links innate and adaptive immunity via crosstalk among neutrophils, monocytes, and T cells.

### 3.2 Post‐Inflammatory Phase

Owing to their essential innate immune function and limited lifespan, a constant production of neutrophils from hematopoietic progenitors occurs in the bone marrow and enters circulation to be recruited by inflammatory signals. Early‐recruited neutrophils undergo apoptosis and are then removed by the M1‐macrophages via phagocytosis, and the multiple‐cells‐mediated inflammatory cascade gradually recedes.

After inflammation resolution, the cytokine level at the defect site decreases to a range that polarizes the later‐arrived neutrophils to the N2‐subtype, which favors bone regeneration. The N2‐neutrophils express a combination of anti‐inflammatory signals (Figure 5B), which transform other immunocytes from proinflammatory types (M1‐macrophage; Th1, Th2, Th17 cells) to anti‐inflammatory types (M2‐macrophage; T_reg_). Distinct sets of cytokines promote the differentiation of CD4^+^ T cells into different lineages: IFN‐*γ* for Th1; IL‐2, IL‐7, TSLP for Th2; IL‐6, IL‐21, IL‐23 for Th17; and TGF*β* for T_reg_.^[^ ^19^ ^]^ As shown in Figure 5B, typical proinflammatory LPS‐activated N1‐neutrophils express IFN‐*γ* and IL‐6, which benefit Th1 and Th17 differentiation, while N2‐neutrophils polarized by specific levels of IL‐8 express TGF*β* for T_reg_. BMSCs also inhibit Th1 and Th17 differentiation and promote the generation of T_reg_ cells,^[^ ^19^ ^]^ which are reported to create and maintain the appropriate immune environment for successful tissue repair.^[^ ^43^ ^]^ Meanwhile, N2‐neutrophils also contribute to macrophage reprogramming toward a reparative M2 phenotype,^[^ ^21^, ^44^, ^45^ ^]^ which later mediate BMSC differentiation by secreting growth factors and inhibiting inflammation.^[^ ^46^ ^]^

Under specific IL‐8 concentrations, N2‐polarized neutrophils release SDF‐1*α* to recruit BMSCs via the SDF‐1/CXCR4 axis and its downstream PI3K/Akt pathway, which consequently lead to *β*‐catenin‐mediated migration (Figure 6B). *β*‐catenin also stimulates fibronectin gene transcription^[^ ^47^ ^]^ to restore ECM components, which had been disturbed by N1‐neutrophil secretions in the inflammatory phase. Directed by the post‐inflammatory regenerative microenvironment delicately orchestrated by both innate and adaptive immunity, BMSCs undergo chondrogenic and osteogenic differentiation to replace the injured bone tissue, commonly known as endochondral ossification.^[^ ^48^ ^]^ Even the slightest imbalance in immunoregulation may lead to unresolved inflammation and sabotage regeneration,^[^ ^49^ ^]^ like the unsupportive microenvironment of the early‐stage fracture hematoma (Figure 1A). Previous design of bone repairing biomaterials emphasizes BMSC recruitment and differentiation by introducing chemoattractants and growth factors.^[^ ^50^ ^]^ However, individual differences in immune microenvironment may lead to failure of even the most potent therapy. With the understanding of bone regeneration initiated by neutrophils, opportunities emerge for biomaterial design to exploit the immunomodulatory function of host neutrophils.^[^ ^3^, ^51^ ^]^

## 4 Conclusion

In this study, we demonstrated the indispensable role of neutrophils at the early stage of bone defect healing and regeneration. IL‐8 implantation first recruited neutrophils, followed by BMSCs and macrophages, and consequently induced rapid ectopic endochondral ossification. Neutrophils were indicated as an upstream participant that recruited and orchestrated innate and adaptive immunocytes, as well as BMSCs, at the initial stage of bone regeneration. IL‐8 was demonstrated as a critical stimulus to alter neutrophil polarization at various concentrations. Under certain IL‐8 levels, neutrophils were N2‐polarized, then secreted SDF‐1*α* for BMSC chemotaxis via the SDF‐1/CXCR4 axis and its downstream PI3K/Akt/*β*‐catenin‐mediated migration. N2‐neutrophils also mediated the anti‐inflammatory phenotype transformation of macrophages and CD4^+^ T cells. The interaction between neutrophil and BMSCs, and innate and adaptive immunities revealed in this study could provide guidance for a new generation of therapeutic biomaterial design that inspires endogenous bone repair. We believe that this work may shed light on the mechanism of neutrophil‐initiated endogenous bone regeneration, arouse broad interests among researchers, and inspire further research into bone repair options.

## 5 Experimental Section

### Materials and Animals

Cell culture related reagents including fetal bovine serum (FBS), *α*‐MEM culture medium, and phosphate buffered solution (PBS) were obtained from Gibco (Thermo Fisher Scientific Inc., MA, USA). Recombinant human IL‐8 (CXCL8, 72 a.a. 200–08M) and SDF‐1*α* were obtained from PeproTech (IL, USA). LPS, IL‐8 ELISA kit, SDF‐1*α* ELISA kit, and DAPI were purchased from Sigma‐Aldrich (St. Louis, MO, USA). The commercial medical absorbable gelatin sponge for IL‐8 loading and fracture hematoma adsorption was obtained from Jiangxi Xiangen medical technology development Co. Ltd, China. Fluorescent‐conjugated monoclonal antibodies for flow cytometry (including PECy5.5‐conjugated anti‐CD45, PECy7‐conjugated anti‐CD29, APC‐conjugated anti‐CD44, PE‐conjugated anti‐F4/80, and FITC‐conjugated anti‐Ly6G) and antibodies for immunohistochemistry (anti‐Col II, anti‐Col I, and anti‐ALP) were purchased from Abcam (Cambridge, UK). All purchased reagents were used without further purification.

All experimental animals (male C57BL/6 mice) were obtained from Laboratory Animal Center of Fourth Military Medical University, Xi'an, China. The animal experiments were performed in strict accordance with the NIH Guide for the Care and Use of Laboratory Animals, and all procedures were carried out with the approval of the Institutional Animal Care and Use Committee of Fourth Military Medical University (Approval No. 2019–079).

### Interleukin‐8 Loading and Releasing

IL‐8 solution was immobilized on a commercial medical absorbable gelatin sponge (IL‐8/gelatin). Briefly, under sterile conditions, the gelatin sponge was cut into 8 × 8 × 5 mm^3^ pieces, then 30 µL of IL‐8 solution containing 5 µg IL‐8 was uniformly dropped onto the gelatin sponge and lyophilized. The IL‐8 dosage was set at 5 µg for an optimized ectopic endochondral ossification in mice as reported in our previous study^[^ ^12^ ^]^ and demonstrated in Figure S1, Supporting Information.

In vitro release profile of IL‐8 from the gelatin sponge was evaluated. Briefly, the IL‐8‐loaded gelatin sponges were placed in test tubes and immersed in 2 mL PBS at 37 °C under a constant shaking of 30 rpm. At each time point, the supernatant was collected and replaced with an equal amount of fresh PBS. The amounts of released IL‐8 were quantitatively analyzed using a human IL‐8 ELISA kit to plot the release profiles.

### Calvarial Defect Creation

8‐week‐old C57BL/6 mice (average weight of 25 g) were anaesthetized with 2% isoflurane inhalation. A linear scalp incision was made from nasal bone to occiput, and full‐thickness flaps were elevated. A 5 mm cranial defect in the center of the parietal calvarial bone with complete resection of the periosteum was created by drilling with trephine under copious irrigation with Hank's balanced salt solution, then the overlying muscle and skin was sutured.

For fracture hematoma acquisition, the gelatin sponge was filled in the created defect before suture, and the mice were euthanized with an overdose of pentobarbital 2 or 4 days post‐operation to retrieve the hematoma‐adsorbed gelatin sponge.

For observation of defect regeneration, the mice were euthanized with an overdose of pentobarbital 90 days post‐operation, and the calvarial samples were obtained for micro‐CT analyses.

### Intramuscular Implantation

Eight‐week‐old male C57BL/6 mice (average weight of 25 g) were anaesthetized with 2% isoflurane inhalation. After shaving and sterilizing, a 5 mm longitudinal incision was made along the hind limb, and a 3 mm deep pocket was created by separating muscle fibers within the biceps femoris. The prepared gelatin sponges were squeezed into the created muscle pouches, then the overlying muscle and skin was sutured. For retrieval of the implants, the mice were euthanized with an overdose of pentobarbital.

### Circulatory Myeloid Cell Depletion

Individual and collective circulatory myeloid cell depletion was realized via neutralizing antibody and radiation‐induced myeloablation, respectively, as previously reported.^[^ ^10^ ^]^

Neutralizing antibodies were administered intraperitoneally for individual depletion of circulating myeloid cells. For neutrophil depletion, rat‐anti‐mouse Ly6G antibody (clone 1A8; Bio X Cell) was administered at 200 µg per mouse 2 days before surgery, and then every other day at 100 µg per mouse until 7 days post‐surgery. For monocyte depletion, rat‐anti‐mouse F4/80 antibody (clone CI:A3‐1; Bio X Cell) was administered at 400 µg per mouse 2 days before surgery, and then every day at 200 µg per mouse until 7 days post‐surgery. Daily injection of rat IgG (200 µg per mouse) served as a control. Monocyte (CD11^+^/F4/80^+^) and neutrophil (CD11^+^/Ly6G^+^) depletion were confirmed in blood samples using flow cytometry (Figure S2, Supporting Information).

Two gamma irradiation sessions (4 Gy dose) with a 4‐day interval were applied for myeloablation. After irradiation, number of monocytes (CD11^+^/F4/80^+^), neutrophils (CD11^+^/Ly6G^+^), and BMSCs (CD45^–^/CD44^+^/CD29^+^) were determined in blood samples using flow cytometry (Figure S4, Supporting Information).

### Myeloid Cell Isolation and Adoptive Transfer

Monocytes, neutrophils, and BMSCs were isolated from the bone marrow of un‐irradiated donor mice and adoptively transferred to irradiated mice via intravenous injection. A mouse neutrophil isolation negative magnetic bead selection kit (BM, Catalog. 480 058, MojoSort, BioLegend) and a mouse monocyte isolation kit (BM, Order no. 130‐100‐629, Miltenyi Biotec) were used for isolation of neutrophils and monocytes, respectively, according to the manufacturer's instructions. Bone marrow stromal cells (BMSCs) were extracted from femur bone marrow. Briefly, both ends of the femur were cut away from the epiphysis, and the bone marrow was flushed out with 15 mL *α*‐MEM culture medium supplemented with 10% FBS and 1% antibiotics (100 U mL^−1^ penicillin G and 100 mg mL^−1^ streptomycin sulphate), then transferred into a 75 cm^2^ polystyrene tissue culture flask and incubated at 37 °C in a humidified atmosphere containing 5% CO_2_. The culture medium was refreshed every 2 days until about 90% confluency was achieved. BMSCs from passage 3–8 were utilized in this study. Purity of the isolated myeloid cells was determined using flow cytometry.

The purified neutrophils, monocytes, and BMSCs from un‐irradiated donor mice were adoptively transferred to the irradiated mice through tail intravenous injection after IL‐8/gelatin implantation. Mice were euthanized with an overdose of pentobarbital 7 days post‐implantation and implants were retrieved for histological analyses.

Further, isolated neutrophils and BMSCs were labeled with fluorescent tracking dyes DiI (Carlsbad, CA, USA) and C34565 (CellTracker Deep Red Dye, Life Technologies), respectively, according to the manufacturer's instructions. Mice were euthanized with an overdose of pentobarbital 2 days after adoptive transfer injection of the fluorescence‐labeled cells and IL‐8/gelatin implantation. Implants were then retrieved for frozen section and fluorescence observation.

### Micro‐CT Analysis

The calvarial specimens were scanned and analyzed using Inveon micro‐CT system (Siemens AG, Germany). Analyses were performed using the manufacturer's evaluation software, the region of the parietal calvarial bone was scanned with a fixed global threshold of 20% (200 on a greyscale of 0–1000). The calvaria were 3D‐reconstructed to quantify the parameters of BMD, bone volume/tissue volume (BV/TV), trabecular number (Tb.N.), trabecular thickness (Tb.Th.), and trabecular separation (Tb.Sp.).

### Implant Cellular Component Analysis

Myeloid cells recruited to the implant were flushed out for analysis. Briefly, 1.5 mL PBS was aspirated using a syringe, which was thrust into the implant to flush out the cells from different directions multiple times until the sample became transparent.

CD45, a positive hematopoietic lineage marker for neutrophils and macrophages^[^ ^52^ ^]^ and negative for BMSCs,^[^ ^53^ ^]^ was chosen for identifying BMSCs, neutrophils, and macrophages in one sample. Thereafter, Ly6G was applied as a specific marker that separated neutrophils from other leukocytes,^[^ ^54^ ^]^ then F4/80 for macrophages.^[^ ^55^ ^]^ Components of myeloid cells were quantified using flow cytometry, with CD45^−^/CD44^+^/CD29^+^ identified as BMSCs, CD45^+^/Ly6G^−^/F4/80^+^ as monocytes, and CD45^+^/Ly6G^+^ as neutrophils. Briefly, each cell suspension group was divided into two equal volumes (one for immunostaining and the other as an unstained control) and centrifuged at 2000 rpm for 5 min for cell collection. The cells were washed and resuspended with PBS, blocked with anti‐FcRII/III antibody for 30 min, and stained with five monoclonal antibodies on ice for 30 min. The immunostained cells and unstained cells were analyzed using a flow cytometer (Beckman Navios, USA) and FLOWJO software (Tree Star, San Carlos, CA, USA). Analysis of each group was performed in triplicate.

### Histology and Immunohistochemistry

The retrieved implants were fixed with 4% paraformaldehyde, dehydrated in a graded series of alcohol, and embedded in paraffin. Then, 4‐µm thick paraffin sections were sliced, deparaffinized, and stained with Masson's trichrome for histological observation and further with safranin‐O for cartilage matrix identification.

For immunohistochemical staining, antigen retrieval was carried out via water‐bath heating with 10 mm sodium citrate buffer. The sections were blocked for 30 min in 10% blocking serum and incubated overnight at 4 °C with primary antibody of Col II, Col I, or ALP. Then the sections were treated with a Streptavidin Biotin Peroxidase Detection kit (Zhongshan, Beijing, China) for color development, according to the manufacturer's protocol, counterstained with hematoxylin and mounted with Permount mounting media.

The slides were observed using a fully automated inverted research microscope (Leica DMI6000 B, Germany). Cell‐wrapped areas around the implant were analyzed using Image J.

### Fluorescence Analysis of Frozen Sections

Retrieved implants were embedded in an optimal cutting temperature compound (SAKURA, USA) on dry ice, then cryosectioned at 10‐µm thickness, followed by DAPI counterstaining (Beyotime, China). Images were captured using confocal laser‐scanning microscopy (CLSM; Leica, Germany).

### RT‐PCR and RT‐PCR Array

Typical inflammatory gene expression of neutrophils treated with various levels of IL‐8 were evaluated using RT‐PCR with a Qiagen master mix (Qiagen, Valencia, CA, USA) and the Bio‐Rad CFX96 real‐time PCR detection system (Bio‐Rad Laboratories, USA) according to the manufacturer's instructions. Mouse inflammatory response and autoimmunity RT^2^ profiler PCR array (Cat. no. 330 231, Qiagen) was applied to further identify the phenotype of neutrophils treated with IL‐8 and LPS. Data were analyzed according to the 2^−∆∆Ct^ method^[^ ^56^ ^]^ and the RT^2^ profiler PCR array data analysis template v4.0 software package (Qiagen). Experiments were performed in triplicate. Primer sequences used in this study are listed in Table S1, Supporting Information.

### Bone Mesenchymal Stem Cell Chemotaxis and Migration Assay

BMSC chemotaxis and migration in the presence of IL‐8, neutrophils, or IL‐8‐treated neutrophils were evaluated in Transwell plates (Corning) with 8 µm porous membrane.

For chemotaxis assays, BMSCs were seeded in the upper chamber with IL‐8 (10 ng mL^−1^), neutrophils, or IL‐8‐treated neutrophils (10 ng mL^−1^, 24 h exposure) seeded in the lower chamber. After 12 h, unmigrated cells on the upper surface of the membranes were gently removed using a rubber scraper; and cells that migrated to the lower surface of the membrane were fixed with 4% paraformaldehyde, stained with crystal violet, and counted in five random fields using an optical microscope (200 ×).

For wound‐healing migration assays, BMSCs were seeded in the lower chamber, with neutrophils or IL‐8‐treated neutrophils (10 ng mL^−1^, 24 h exposure) seeded in the upper chamber. A linear wound was scratched using a pipette tip after adherence of BMSCs. Wound closure of BMSCs after 24 h was observed and imaged using an optical microscope.

To investigate the role of the SDF‐1/CXCR4 axis and PI3K/AKT pathway in BMSC chemotaxis toward neutrophils, prior to the migration assay, BMSCs were incubated with SDF‐1*α*/CXCR4 cascade antagonist AMD3100 (100 µM; Selleck Chemicals, Houston, TX, USA) for 1 h or PI3K inhibitor LY294002 (20 µM; Abcam, Cambridge, MA, USA) for 2 h.

### Cytokine Protein Array

Secreted cytokines from neutrophils and IL‐8‐treated neutrophils were evaluated using mouse cytokine antibody array (ab133995, Abcam, Cambridge, MA, USA) according to the manufacturer's instructions. Antigen‐antibody complexes were visualized using LumiGLO substrate (Kirkegaard & Perry Laboratories, Inc.) and chemiluminescent sensitive film (Kodak). Densitometry was performed via image analysis (Image J) for quantification of cytokine secretion.

### Western Blot

BMSCs were treated with SDF‐1*α* (100 ng mL^−1^; Proteintech Group, Chicago, IL, USA) for 30 min. Total cellular proteins were extracted using RIPA lysis buffer containing 1 mm phenylmethanesulfonyl fluoride. The extracted proteins were separated via 12% SDS‐PAGE and transferred to a polyvinylidene fluoride membrane. The membrane was incubated in Tris buffered saline with tween 20 containing 5% bovine serum albumin (BSA) for 1 h at room temperature (24–26 °C). Afterward, the membranes were incubated with antibodies against AKT and p‐AKT (Abcam, Cambridge, MA, USA) at 4 °C overnight, followed by peroxidase‐conjugated AffiniPure goat anti‐rabbit IgG (H+L) for 1 h at room temperature. Immunoreactivity was determined using chemiluminescence. The relative integrated density values were measured using GAPDH as a control. To block and verify the potential signaling pathway, BMSCs were incubated with AMD3100 for 1 h or LY294002 for 2 h before SDF‐1*α* treatment.

### Immunofluorescent Staining

For fluorescent staining of the migration‐related protein *β*‐catenin, after 30 min treatment with SDF‐1*α*, BMSCs were fixed with 2.5% glutaraldehyde, permeabilized with 0.1% Triton X‐100 solution for 15 min and incubated with 5% BSA for 1 h to block non‐specific binding. Thereafter, cells were incubated with an antibody against *β*‐catenin (Abcam, Cambridge, MA, USA) at 4 °C overnight, followed by FITC‐labeled goat‐anti‐rabbit IgG (Abcam) for 2 h at room temperature. Cell nuclei were identified with DAPI. CSLM was used to examine the expression and distribution of *β*‐catenin.

### Statistics Analysis

Results were expressed as mean ± standard deviations. All data were generated using at least three independent experiments. Statistical analysis was conducted using one‐way analysis of variance and Tukey's post hoc test. A value of *p* < 0.05 was considered statistically significant. Statistical Product and Service Solutions (SPSS 21.0) software was used for statistical analysis.

## Conflict of Interest

The authors declare no conflict of interest.

## Supporting information

Supporting Information

## Data Availability

Research data are not shared.
