# Supplementary material for: N2‐Polarized Neutrophils Guide Bone Mesenchymal Stem Cell Recruitment and Initiate Bone Regeneration: A Missing Piece of the Bone Regeneration Puzzle
Source: Adv Sci (Weinh). 2021 Aug 11;8(19):2100584. doi: 10.1002/advs.202100584 (PMC8498914; doi:10.1002/advs.202100584)
Supplement: Supplementary file 1 — Supporting Information [file ADVS-8-2100584-s001.pdf]

© 2021 Wiley-VCH GmbH

ADVANCED SCIENCE
Open Access

Supporting Information

for *Adv. Sci.,* DOI: 10.1002/advs.202100584

N2-polarized neutrophils guide BMSC recruitment and initiate bone regeneration: A missing piece of the bone regeneration puzzle

Bolei Cai, Dan Lin, Yan Li, Le Wang, Jirong Xie, Taiqiang Dai, Fuwei Liu, Mingyue Tang, Lei Tian, Yuan Yuan*, Liang Kong*, and Steve GF Shen**

Supporting Information

N2-polarized neutrophils guide BMSC recruitment and initiate bone regeneration: A missing piece of the bone regeneration puzzle

Bolei Cai, Dan Lin, Yan Li, Le Wang, Jirong Xie, Taiqiang Dai, Fuwei Liu, Mingyue Tang, Lei Tian, Yuan Yuan*, Liang Kong*, Steve GF Shen**

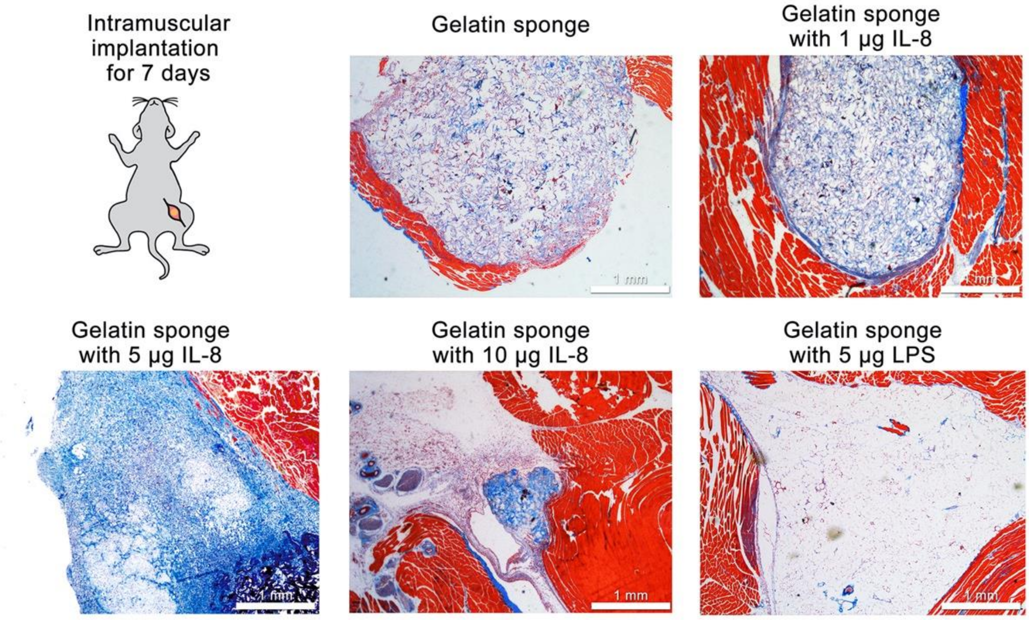

Figure S1. Intramuscular ectopic implantation of gelatin sponge, gelatin sponge with 1 μg IL-8, 5 μg IL-8, 10 μg IL-8, and 5 μg LPS. Gelatin sponge with a low IL-8 dose (1 μg) was insufficient to recruit myeloid cells and induce endochondral ossification, while a high IL-8 dose (10 μg) led to the rapid degradation of gelatin, indicating predominant catabolism. The gelatin sponge with LPS was completely degraded and pathological fat liquefaction was observed at the implantation site (n = 3 for each group).

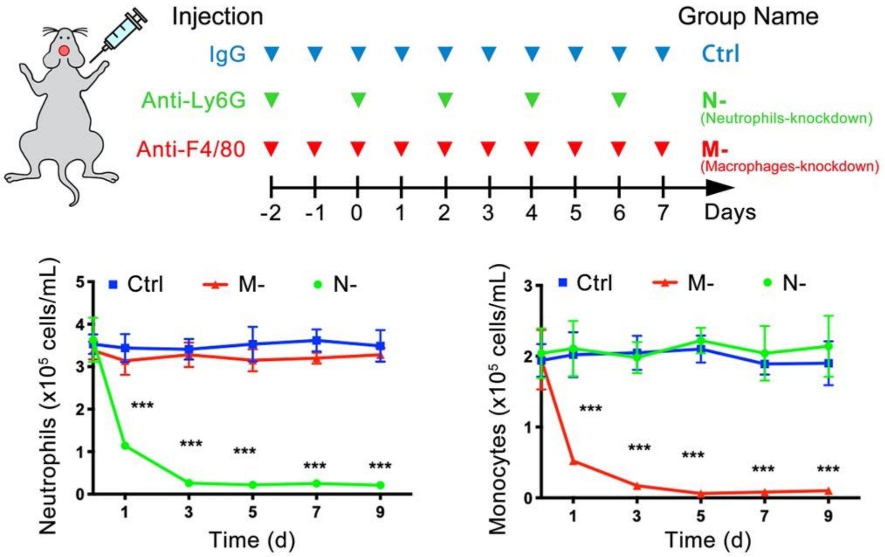

Figure S2. Time-course of circulatory neutrophils and monocytes quantified using flow cytometry after treatment with anti-Ly6G or anti-F4/80 neutralizing antibodies. The

neutralizing antibodies efficiently and specifically depleted neutrophils or monocytes in peripheral blood without affecting other cell type (n = 3 for each group).

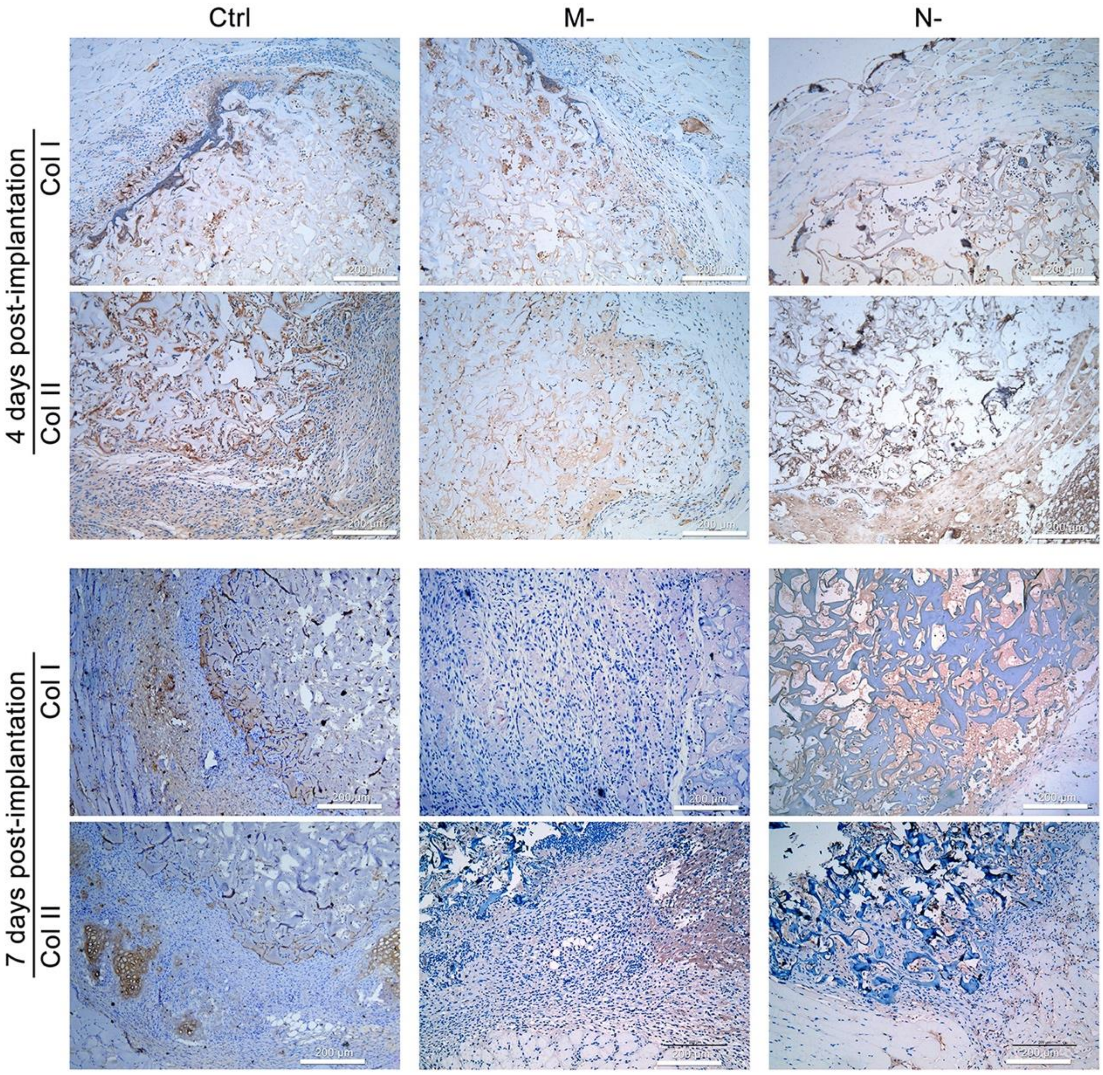

Figure S3. Immunohistochemical staining of Col I and Col II in the ectopic tissue after depletion of neutrophils/macrophages (N-/M-). Neutrophil/macrophage depletion significantly hindered chondrogenic and osteogenic differentiation around the implantation site (n = 3 for each group).

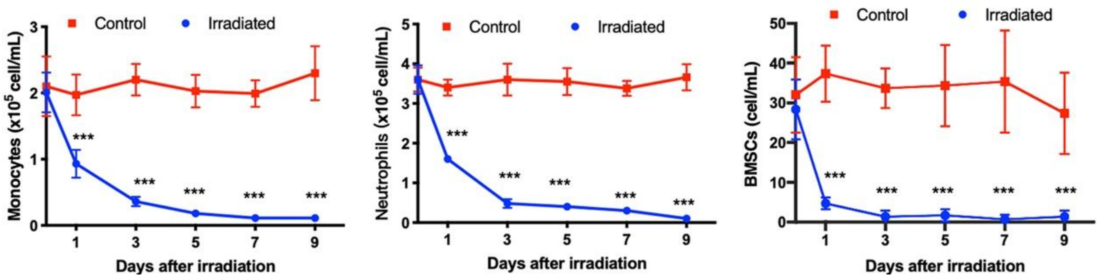

Figure S4. Time-course of circulatory myeloid cells in nonlethal irradiated mice quantified using flow cytometry. The number of myeloid cells in peripheral blood decreased by over 90% three days post-irradiation (n = 3 for each group).

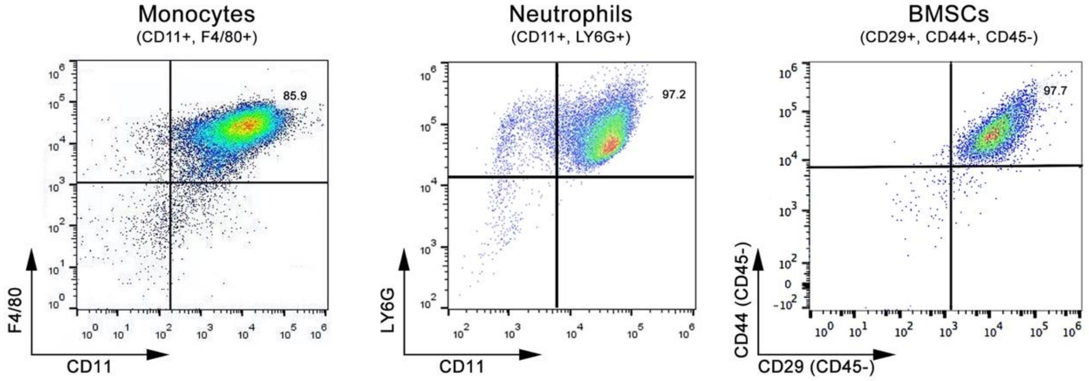

Figure S5. Flow cytometry analysis of isolated myeloid cells from unirradiated mice. The positive rates of the purified monocytes, neutrophils, and BMSCs were 85.9%, 97.2%, and 97.7%, respectively, which met the requirements of subsequent experiments.

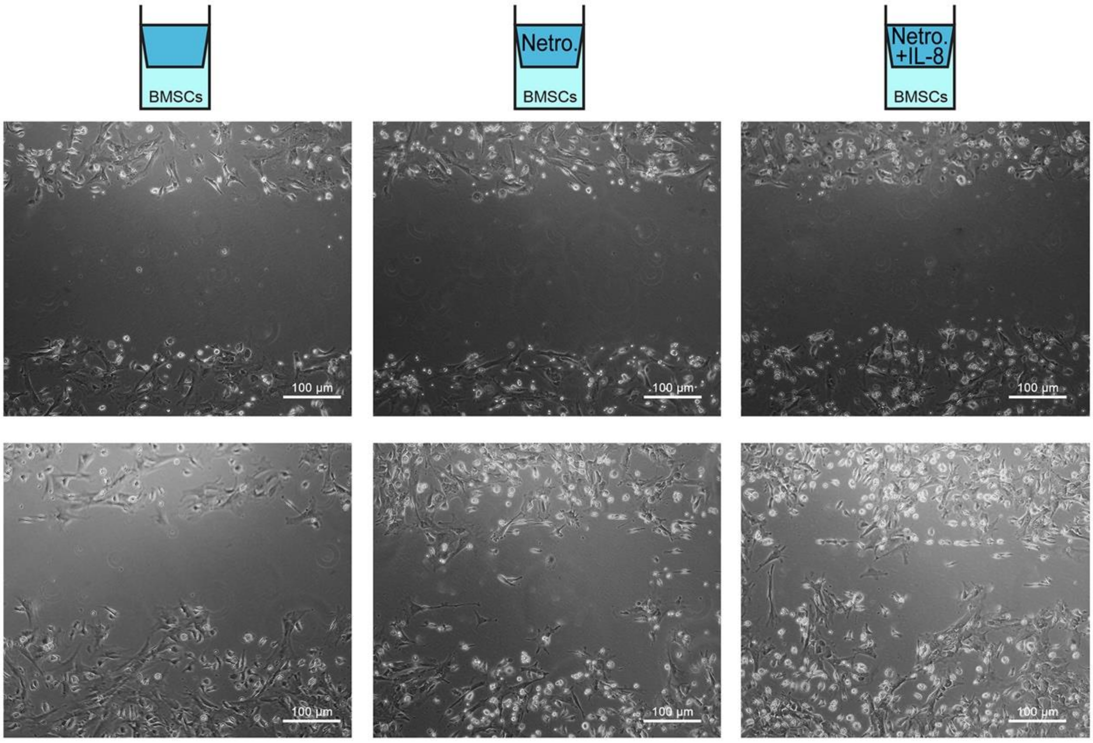

Figure S6. Wound-healing assay of BMSC migration co-cultured with neutrophils or IL-8-treated neutrophils for 24 h in transwell. IL-8-treated neutrophils significantly enhanced the migration capacity of BMSCs.

Table S1. Primer sequences used in RT-PCR.

Primer	Sequences
mouse IL-10 Forward	GGTTGCCAAGCCTTATCGGA
mouse IL-10 Reverse	GGGGAGAAATCGATGACAGC
mouse TGFβ1 Forward	CTTCAATACGTCAGACATTCGGG
mouse TGFβ1 Reverse	GTAACGCCAGGAATTGTTGCTA
mouse TNF Forward	GGCAGGTCTACTTTGGAGTC
mouse TNF Reverse	TCGAGGCTCCAGTGAATTCG
mouse IFNγ Forward	AACGCTACACACTGCATCTTG
mouse IFNγ Reverse	GACTTCAAAGAGTCTGAG
